# Voice Tremor and Botulinum Neurotoxin Therapy: A Contemporary Review

**DOI:** 10.3390/toxins14110773

**Published:** 2022-11-09

**Authors:** David P. Newland, Daniel Novakovic, Amanda L. Richards

**Affiliations:** 1 Department of Otolaryngology, The Royal Melbourne Hospital, Parkville, VIC 3050, Australia; 2 Voice Research Laboratory, Faculty of Medicine and Health, University of Sydney, Camperdown, NSW 2050, Australia; 3 Department of Otolaryngology, The Canterbury Hospital, Campsie, NSW 2194, Australia

**Keywords:** voice tremor, essential tremor, botulinum neurotoxin

## Abstract

Voice tremor is a common, yet debilitating symptom for patients suffering from a number of tremor-associated disorders. The key to targeting effective treatments for voice tremor requires a fundamental understanding of the pathophysiology that underpins the tremor mechanism and accurate identification of the disease in affected patients. An updated review of the literature detailing the current understanding of voice tremor (with or without essential tremor), its accurate diagnosis and targeted treatment options was conducted, with a specific focus on the role of botulinum neurotoxin. Judicious patient selection, following detailed characterisation of voice tremor qualities, is essential to optimising treatment outcomes for botulinum neurotoxin therapy, as well as other targeted therapies. Further focused investigation is required to characterise the response to targeted treatment in voice tremor patients and to guide the development of innovative treatment options.

## 1. Introduction

Affecting 1–5% of the general population, essential tremor (ET) is the most common movement disorder [1]. Despite being so common, because ET is a clinical diagnosis and the spectrum of tremor-associated disorders frequently overlap [2,3,4], this poses challenges for the treating clinician and therefore typically makes ET a diagnosis of exclusion. In 2018, the International Parkinson and Movement Disorder Society (IPMDS) proposed updated diagnostic criteria for ET, including the following four features—isolated tremor consisting of bilateral upper limb action tremor without other motor abnormalities; present for at least three years; with or without head, voice or lower limb tremor; absence of other neurologic signs such as dystonia, ataxia or parkinsonism. Isolated voice tremor is now removed from the essential tremor classification [5]. As such, studies pre-reclassification will be herewith referred to with nomenclature that is appropriate to the period in which they were classified.

Essential voice tremor (EVT), previously described as the laryngological manifestation of ET, is present in 18–30% of ET patients [6], as the third most common tremor site after the hands and head [7]. EVT typically presents with tremor associated with increased phonatory effort [8]. Prior to reclassification, studies report that EVT may be present in the absence of any other manifestations of ET [9]; however, a more recent consensus statement proposed updated diagnostic criteria which state that isolated voice or head tremor are not sufficient for a diagnosis of ET [5]. ET is independently associated with the female gender [10,11]. The disease may be familial—showing an autosomal-dominant inheritance pattern with reduced penetrance—or sporadic, but no key neurophysiologic or monogenic form has been identified [2,12,13]. Genome-wide association studies (GWAS) have identified an association between a genetic polymorphism involving the gene that encodes the LINGO1 protein [14], which plays important roles in inhibiting cell differentiation, axonal regeneration and synaptic plasticity [4]. The peak onset of ET occurs around the fifth to sixth decades of life and symptom progression typically occurs with age [4,7,12]; however, familial and alcohol-responsive forms of ET may exhibit an earlier onset [2]. While symptoms typically worsen gradually, they may be exacerbated by other physiological or psychological stressors, including exercise, fatigue or stress.

The underlying pathophysiology of ET involves both cerebellar and brainstem dysfunction [15]. This is supported clinically in the manifestation of non-tremor symptoms of ET including gait ataxia, oculomotor abnormalities, mood disturbance and motor learning issues [16]. Neurodegenerative changes occur in the brainstem and cerebellar dentate nucleus [17], with consequent GABAergic dysfunction involving the cerebellum and locus coeruleus and subsequent tremulous activity within the cerebello-thalamo-cortical circuit has been identified [18]. Pathologic Purkinje cell axonal swellings representing damage to Purkinje cell axons have been identified both in the cerebellar vermis and hemispheres [7,10]. As previously described, understanding the pathophysiology of EVT requires consideration of the interplay between the intrinsic disease state and the central neural pathways involved in voice production, as well as the modulatory roles of oral, pharyngeal and pulmonary structures [7,11]. While differences between the neural pathways involved in innate and learned vocalisation are recognised, there is involvement of the primary motor area, superior temporal gyrus, anterior insular cortex, anterior cingulate cortex, basal ganglia, periaqueductal gray and cerebellum [19]. Specifically, structural neuroimaging and post-mortem histopathological studies of head and voice tremor patients demonstrate increased atrophy of the cerebellar vermis, consistent with known cerebellar somatotopic organisation [16,20]. However, more widespread neurodegenerative changes affecting both white and grey matter in many cortical, subcortical and brainstem structures have also been described in ET [15]. It was previously proposed that head and voice tremor may represent distinct subtypes of ET [10], and this is reflected in the updated classification.

The clinical assessment of the tremor patient should rely on characterisation of tremor phenomenology—either in isolation, or in combination with other movement disorders. Key features of the history in patients with suspected ET include the age of onset and family history of tremor, dystonia, parkinsonism, ataxia and dementia [2,21]. Voice complaints are frequently an early presenting symptom of neurological disorders [22].

## 2. Characteristics of Tremor

Understanding tremor aetiology involves detailed characterisation of its qualities. These include the frequency and distribution of the tremor, whether there is unilateral or bilateral involvement, the presence of rest, postural and kinetic elements of the tremor, and whether the tremor exhibits task specificity. The regular tremor of ET characteristically ranges from 4 to 10 Hz; however, significant variability may exist [11,23]. Compared with the predominantly resting tremor of Parkinson disease, ET has both greater postural and kinetic elements; however, 20% of ET patients exhibit resting tremor [11]. Unlike spasmodic dysphonia (SD), EVT is both regular and directionally symmetric [24]. Anatomically speaking, EVT may be produced by multiple different sources, including the articulatory system (including the jaw, tongue and lips), the respiratory system and the phonatory system [7,25], which may reveal tremor in up to 40% of ET patients [26], and it should be noted that head and voice tremor also frequently co-exist. Close attention should be paid to the neck, particularly the extrinsic laryngeal musculature [7]. Under flexible transnasal laryngoscopic assessment, in the awake, unsedated patient without topical anaesthesia, respiration, sustained vowel phonation, connected speech and maximum phonation time are assessed [7,27]. Patients with EVT are typically affected at multiple laryngeal subsites, with large-series data demonstrating true vocal fold (horizontal) tremor in 100%, supraglottic tremor in 95% and global laryngeal (vertical) tremor in 85% of cases [7,22]. Palatal, tongue base and pharyngeal wall involvement are also frequently seen. Characterising both the location and directionality of the tremor is important in EVT, and vocal tremor severity correlates with increasing subsite involvement, tremor severity of the supraglottis and vertical laryngeal movement [7,26].

## 3. Tools for Detailed Investigative Analysis of Essential Voice Tremor

Both patient self-evaluation of their voice using a self-administered questionnaire such as the abbreviated Voice Handicap Index (VHI-10) [28] and use of a validated outcome measure for perceptual evaluation such as the Voice Perceptual Profile [29] or the Consensus Auditory-Perceptual Evaluation of Voice (CAPE-V) questionnaire [30] will allow for pre- and post-treatment assessment. Rating scales such as the Vocal Tremor Scoring System (VTSS) [31] and acoustic analysis [32] allow for detailed analysis of the site and severity of the tremor. The VTSS details the severity of tremor across six anatomic subsites based on flexible transnasal laryngoscopic assessment—palate, tongue base, pharyngeal wall, larynx globally, supraglottis and true vocal folds [31]. Specifically, it defines the following: “pharyngeal wall tremor consists of rhythmic medialisation of the lateral wall of the pharynx”; “global laryngeal tremor refers to motion in the vertical dimension of the larynx…relative to the surrounding aerodigestive tract”; “supraglottic tremor…refers to tremor in the anterior-posterior and lateral dimensions of the epiglottis, ventricular folds, aryepiglottic folds and supraglottic portion of the arytenoids”; “true vocal fold tremor refers to activity in the glottal plane consisting of oscillation of the vocal folds in the lateral dimension” [31].

Neurophysiological assessment often plays an important adjunctive role in guiding the treatment of voice disorders and is typically performed using laryngeal electromyography (EMG). Laryngeal EMG involves the use of electrodes to assess the neuromuscular activity of the larynx by recording action potentials within its intrinsic and extrinsic musculature [33]. The electrodes used may be either non-invasive surface electrodes or percutaneous needle electrodes. The electrodes are typically placed using anatomical landmarks and their position verified based on characteristic EMG activity with specific laryngeal activity [33]. The most commonly tested muscles are cricothyroid (CT), thyroarytenoid (TA) and posterior cricoarytenoid (PCA). Laryngeal EMG demonstrates findings of contractile activity during both active and passive laryngeal tasks [9], but may not reliably differentiate EVT from other causes of tremor and is not routinely performed for diagnostic purposes.

## 4. Treatment Options for Essential Voice Tremor

The treatment options for essential voice tremor may be classified as oral pharmacological, non-oral pharmacological and non-pharmacological.

### 4.1. Oral Pharmacological Treatment Options for Essential Voice Tremor

Oral medications are often considered first line in the treatment of EVT. These include propranolol, a non-selective beta-adrenergic receptor antagonist, and primidone, a benzodiazepine. Propranolol acts to reduce adrenergic activity and its lipophilic profile allows a better central effect on the tremor [34], although voice tremor response to propranolol is variable. In a comparison of the effectiveness of onabotulinum toxin Type A and propranolol for EVT [35], 56% of patients reported an improvement in their voice-related quality of life (VRQOL) whilst on propranolol. However, when compared to onabotulinum toxin Type A, propranolol has a lesser effect on VRQOL. The main adverse effects associated with propranolol use include bronchospasm and bradycardia, so careful consideration must be given to their use in patients with comorbid cardiac and respiratory disease. Primidone acts to increase gamma-amino butyric acid (GABA)-A activity and is particularly suited to patients with alcohol-responsive disease, with a response rate in the order of 50% for EVT [7,8]. Owing largely to its common side effects, including fatigue, nausea and malaise, the tolerance of primidone therapy is variable [8]. Other oral medications that have been trialled in the treatment of EVT include methazolamide [36], a carbonic anhydrase inhibitor, and more recently, octanoic acid [37], a derivative of long-chain alcohol 1-octanol—despite showing significant effects on frequency modulation and tremor amplitude, respectively, neither have demonstrated significant subjective symptomatic improvement based on patient-reported outcomes.

### 4.2. Non-Oral Pharmacological Treatment of Essential Voice Tremor

The non-oral pharmacological treatment of EVT involves the use of botulinum neurotoxin (BoNT) injected to a number of different anatomic subsites under laryngeal EMG guidance. While Jankovic and Schwartz first reported the effects of BoNT type A (BoNT-A) treatment in essential tremor in 1991 [38], the first published reports of BoNT-A treatment for EVT were not until 2000 [39]. As previously described, the use of BoNT-A for the treatment of EVT is more effective than propranolol when assessed using a VRQOL questionnaire [35]. More recently, BoNT has become considered part of the standard of care for EVT; however, it should be noted that EVT from palatal, tongue and pharyngeal involvement is considered less responsive to BoNT. The distribution of laryngeal EMG-guided BoNT injections must be correlated to both the direction and location of the tremor in order to achieve the most successful treatment outcome. Whilst glottic incompetence is a common feature of EVT, early data suggest no advantage of augmentation injection laryngoplasty compared with BoNT-A [40].

#### 4.2.1. Botulinum Neurotoxin Formulations

It is important to recognise that other formulations of BoNT type A and BoNT type B exist; however, their commercial availability is subject to a number of additional factors including the licencing and certification of the different formulations by the relevant regulatory body. For example, there are three commercially available BoNT-A products in Australia—onabotulinum toxin Type A (Botox^®^, Allergan, Irvine, CA, USA), abobotulinumtoxin Type A (Dysport^®^, Galderma Laboratories, L.P, Fort Worth, TX, USA) and, more recently, incobotulinumtoxinA (Xeomin^®^, Merz Pharmaceuticals GmbH, Frankfurt, Germany). While the use of all three of these formulations has been described in the treatment of spasmodic dysphonia [41], there are no published reports of Xeomin use for treatment of essential voice tremor. With this in mind, it is important to remember that spasmodic dysphonia and essential voice tremor represent different underlying pathophysiology, so the response to treatment with different BoNT-A formulations may differ and should not be extrapolated. There are currently no commercially available formulations of BoNT type B in Australia, nor are there published reports of its use in the treatment of essential voice tremor.

Each formulation has its own unique pharmacology and doses are not interchangeable. In line with the majority of published series examining the treatment of EVT with BoNT-A describing use of Botox^®^, our neurolaryngology clinic also primarily uses Botox^®^, while Dysport^®^ is typically reserved for cases that demonstrate resistance to onabotulinum toxin type A use. The dose equivalence of Dysport^®^ to Botox^®^ is widely debated, but it is considered to have a ratio of between 2.5:1 and 3:1 [42]. Another consideration to guide BoNT-A formulation selection may be the differential spread of toxin from its site of injection, with Dysport^®^ considered to demonstrate significantly greater spread than Botox^®^ in dermatologic applications [42].

#### 4.2.2. Botulinum Neurotoxin Injection Technique

The authors use onabotulinum toxin Type A (Botox^®^, Allergan, Irvine, CA, USA) made to a standard solution of 50 units (U) per 1 cubic centimetre (cc) by adding 2 cc of sterile normal saline to a 100 U bottle of powdered BoNT-A. As our neurolaryngology clinic sees patients with a combination of laryngeal dystonia and essential/isolated voice tremor, this dilution allows for the larger dose range that is required in tremor patients whilst maintaining a volume that allows for targeted delivery. Sterile injectable saline then dilutes the injectate to the desired concentration and the desired dose is delivered in a 1 cc graduated luer lock syringe.

Accurate localisation of target muscle is achieved through use of laryngeal EMG. The most basic setup utilises a handheld, single-channel EMG with acoustic feedback. Electrical interference should be minimised during the procedure. A 1.5 inch, 26- or 27-gauge, Teflon-coated therapeutic needle (monopolar electrode) is suitable for both diagnostic EMG, when indicated, along with therapeutic injections. In the absence of EMG, endoscopically-guided injections may be utilised for the thyroarytenoid muscle [43].

The patient is positioned on a bed or chair in a semi-reclined position with a pillow to allow for neck extension. Ground and negative references are attached to the patient’s skin over the clavicle and sternocleidomastoid muscle, respectively, and connected to the EMG machine. 

#### 4.2.3. Consent/Patient Information Prior to Botulinum Neurotoxin Treatment

Pre-procedure counselling occurs in the clinician’s office, prior to the procedure. While discussing the procedure details, it is emphasised that the BoNT treatment offers symptomatic relief by temporary chemodenervation without cure. BoNT dosage may vary over time depending upon disease activity [44], and the patient is counselled that determining optimum dosing may require several cycles of treatment. Consent includes discussion of common and expected side effects, specifically breathiness and dysphagia, especially to liquids [45]. Patients are asked to record the nature, severity and duration of side effects to guide future doses and dosing intervals. Our clinic performs telephone follow-up at 4 weeks post injection, utilising percentage of normal function scores along with the VHI-10 to assess treatment outcome. In the context of the COVID-19 pandemic, patient-initiated voice recordings have been utilised for expert listener ratings, rather than face-to-face reviews. Patients should be reassured that they can discontinue treatment at any time without ill effect, whereby the voice will return to baseline, and that they will not develop a physical “dependence” upon the BoNT treatments, which is a common misconception.

#### 4.2.4. Botulinum Neurotoxin Treatment of Specific Types of Voice Tremor

For our voice tremor treatments, the desired dose and concentration of BoNT-A is made by utilising 5 units per 0.1 cc of sterile normal saline for injection and drawing up into a 1 cc luer lock syringe, with further saline dilution reflecting specific muscle requirements. Dependent on injector preferences, excess BoNT-A may be discarded from the syringe prior to usage to prevent accidental overdosing.

##### Botulinum Neurotoxin Treatment for Horizontal Glottic Tremor

Horizontal glottic tremor is typically managed with thyroarytenoid/lateral cricoarytenoid muscle complex injections. Owing to the reliable diffusion from TA muscle injections to lateral cricoarytenoid (LCA) in the majority of cases [46], they may be treated as a single injection target [47]. Of those that specify BoNT-A injection technique for TA/LCA in EVT, all express a preference for a percutaneous transcricothyroid space approach through the cricothyroid membrane under EMG guidance [9,39,40,45,48,49,50]. Endoscopically-assisted transoral and percutaneous BoNT-A TA injections are described in the treatment of laryngeal dystonia, but not in the EVT literature. The needle is inserted via the cricothyroid space about 3 mm lateral to the midline and directed 30 to 45 degrees superiorly and laterally. The EMG machine is activated, and the needle advanced below the inferior lip of the thyroid cartilage into the paraglottic space, yielding crisp sharp EMG potentials when the tip of the EMG needle is near the motor endplates of the TA muscle. The patient is then asked to phonate gently at fundamental frequency for EMG confirmation of the position of the needle before injecting. Typically, a 0.1 cc aliquot is used into each vocal fold at the desired concentration. Both unilateral and bilateral TA BoNT-A injections are described in the EVT literature, but the majority of patients receive bilateral treatment. Examining the response to unilateral, compared with bilateral, TA BoNT-A injection for treatment of EVT, Warrick et al. found no significant difference in treatment outcomes by acoustic analysis or subjective patient reported measures [48]. Others favour bilateral treatment in view of the understanding that EVT is a bilateral, directionally symmetric pathology [40]. Botox^®^ dosing for TA injections in EVT ranges from 0.5 to 15 units [9,39,40,45,48,49], while Guglielmino et al. describes the use of 15 units Dysport^®^ [50]. Our institutional practices involve initial dosing of 1 unit Botox^®^ bilaterally, or 2.5 units unilaterally, depending on clinician.

##### Botulinum Neurotoxin Treatment for Vertical (Global) Laryngeal Tremor

A predominantly vertical component to the tremor warrants bilateral infrahyoid strap muscle BoNT-A injection in order to minimise the vertical movement of the larynx. Percutaneous BoNT-A injection of the infrahyoid strap muscles is performed without the use of EMG guidance with the needle directed laterally away from the midline at a level corresponding to the midpoint of the thyroid lamina bilaterally—this technique relies on the use of anatomical landmarks. Due to the proximity of BoNT-A injections to the hyoid and associated diffusion into the tongue base, strap muscle injection may be associated with greater risk of dysphagia [9]. Botox^®^ dosing for strap muscle injections in EVT ranges from 2 to 5 units to each side initially [9,49]. Compared with BoNT-A injection for horizontal glottic tremor, in our experience, higher initial doses of 10 to 15 units BoNT-A to each side are used and may be delivered as a multi-level injection.

##### Botulinum Neurotoxin Treatment for Mixed Horizontal and Vertical Laryngeal Tremor

In line with the approach proposed by Gurey et al. [9], in cases of EVT with mixed horizontal and vertical components, our practice is to stage treatment with a staggered approach to reduce the risk of dysphagia and aspiration. However, Nelson et al. has quantitatively demonstrated the benefits of concurrent bilateral TA and strap muscle BoNT-A injection for patients with mixed horizontal and vertical laryngeal components to their essential voice tremor, with improved post-treatment VHI-10 and CAPE-V scores and comparable rates of associated dysphagia and breathiness [49]. The perceptual profile determines the dominant feature. Increasingly severe phonation breaks, interrupting fluency, guide the thyroarytenoid as the starting point, whereas a rhythmic tremor with mild or slight phonation breaks only directs us to initially target the infrahyoid strap muscles.

#### 4.2.5. Limitations of Botulinum Neurotoxin Treatment for Voice Tremor

While BoNT treatment for voice tremor may be more effective than oral medications, the effects remain temporary and therefore must be continually repeated in order for patients to receive therapeutic benefit. With such limited and heterogeneous published data describing the durability of BoNT treatment for EVT, the frequency with which BoNT injections must be repeated remains unclear. With this in mind, consideration must be given to the cumulative risks and adverse effects, financial burden and impact on quality of life associated with the need for repeated BoNT injections.

### 4.3. Treatment of Medically-Refractory Essential Voice Tremor

Second line treatments for medically-refractory EVT include neurostimulatory and ablative options. Both of these treatment options seek to achieve a degree of neuronal inhibition in either the ventral intermediate nucleus (Vim) of the thalamus [23,51] or the caudal zona incerta (cZi) of the basal ganglia [3].

#### 4.3.1. Deep Brain Stimulation for Treatment of Essential Voice Tremor

Deep brain stimulation (DBS) involves surgically implanted electrodes that target different locations within the thalamus and basal ganglia [3]. DBS was first employed as a treatment for essential tremor in 1997 [52] and remains the mainstay of treatment for patients with medically-refractory ET, favoured over the permanent effects of ablative techniques due to its reversible and adjustable nature. DBS may either be unilateral or bilateral. Individual responses to DBS vary and reduction in vocal tremor may be seen in 50 to 95% of patients [3,53]. Stimulation of Vim versus cZi may have differing ET treatment outcomes [54]; however, there is limited evidence directly comparing any difference in treatment response in EVT.

Adverse effects of DBS include dysarthria, sensory disturbances, and gait and balance disturbances; however, these are typically reversible [3,4,55,56]. Adverse effects are more common with bilateral DBS compared with unilateral DBS [57,58].

A proportion of patients treated with DBS for ET also experience waning treatment effects, with multiple postulated mechanisms, including the development of tolerance to neurostimulation and disease progression [59,60].

#### 4.3.2. MRI-Guided Thalamotomy for Essential Voice Tremor

Prior to the development of DBS, ablative neurosurgical procedures have been used since the 1950s to create a thalamic or subthalamic lesion for the effective treatment of tremor [61,62]. In 2016, a randomised controlled trial involving 76 participants described the results of the creation of a permanent, unilateral, MRI-guided focused ultrasound (MRgFUS) thalamotomy using focused ultrasound in patients with moderate to severe ET [61]. Subsequent trials have confirmed similar findings [63,64]. Using the validated systems such as the Clinical Rating Scale for Tremor (CRST), the benefits of MRgFUS thalamotomy for ET are sustained for at least 2 years [65,66,67]. Indeed, a recent systematic review found that MRgFUS demonstrates greater improvement in quality of life measures, despite lesser improvement in tremor severity scores compared with DBS [68]. However, just as for DBS, there is very limited published evidence detailing the response of EVT to MRgFUS thalamotomy or with specific focus on vocal tremor outcomes. The most common adverse effects of thalamotomy include gait disturbance and sensory disturbance [66].

## 5. Conclusions

Voice tremor continues to prove an incapacitating symptom for patients. Our ability to target effective treatments relies on a nuanced understanding of the underlying pathophysiology, which continues to be refined. Further investigation is required to optimise our understanding of the disease process in order to improve treatment outcomes for patients with voice tremor.

## Data Availability

Not applicable.
